# Community responses to seawater warming are conserved across diverse biological groupings and taxonomic resolutions

**DOI:** 10.1098/rspb.2017.0534

**Published:** 2017-09-06

**Authors:** Dan A. Smale, Joe D. Taylor, Steve H. Coombs, Gerald Moore, Michael Cunliffe

**Affiliations:** 1 Marine Biological Association of the United Kingdom, The Laboratory, Citadel Hill, Plymouth PL1 2PB, UK; 2 Department of Biology, University of York, Wentworth Way, York YO10 5DD, UK; 3 Bio-Optika, Gunnislake PL18 9NQ, UK; 4 Marine Biology and Ecology Research Centre, School of Biological and Marine Sciences, Plymouth University, Drake Circus, Plymouth PL4 8AA, UK

**Keywords:** assemblage composition, benthic invertebrates, beta diversity, community dynamics, ocean warming, marine biofilms

## Abstract

Temperature variability is a major driver of ecological pattern, with recent changes in average and extreme temperatures having significant impacts on populations, communities and ecosystems. In the marine realm, very few experiments have manipulated temperature *in situ*, and current understanding of temperature effects on community dynamics is limited. We developed new technology for precise seawater temperature control to examine warming effects on communities of bacteria, microbial eukaryotes (protists) and metazoans. Despite highly contrasting phylogenies, size spectra and diversity levels, the three community types responded similarly to seawater warming treatments of +3°C and +5°C, highlighting the critical and overarching importance of temperature in structuring communities. Temperature effects were detectable at coarse taxonomic resolutions and many taxa responded positively to warming, leading to increased abundances at the community-level. Novel field-based experimental approaches are essential to improve mechanistic understanding of how ocean warming will alter the structure and functioning of diverse marine communities.

## 1. Introduction

Understanding community-level responses to environmental change is a central goal of ecology. Recent alterations in environmental conditions, principally caused by human activities, have rendered this goal more pressing since a better understanding of ecological processes is necessary to improve our ability to detect and predict the impact of future changes [1,2]. The majority of experimental work on the effects of both climate [3–5] and non-climate [6,7] stressors on multi-species assemblages has been conducted under controlled conditions, in mesocosm systems for example, where environmental variables can be manipulated more readily. The choice of target organism(s) in controlled experiments is, however, heavily prejudiced by the investigator and rarely represents natural or complete communities, while tightly-controlled ecological experiments may also suffer from artificiality [8–10]. As such, the influence of environmental variability on complex communities representing naturally-occurring species pools has seldom been examined [8]. This limitation currently restricts the inference space of experimental ecology, particularly with regards to marine ecosystems. Using novel technology and approaches to translocate manipulative experiments from the laboratory to natural field settings will increase realism and, to a large extent, remove issues of artificiality and representativeness [8,10].

Climate change is driving the redistribution of species, reorganization of communities and restructuring of entire ecosystems at global scales [11,12]. Warming, through both gradual increases in temperature and short-term extreme events (i.e. heat waves) influences processes across all biological scales and can lead to step-wise shifts in ecosystem structure and functioning [13]. In the marine realm, temperature has long been recognized as a fundamental factor influencing the biology of ectotherms [14–16], and recent ocean warming trends have had both direct and indirect effects on populations [17], communities [18] and ecosystems [19]. Understanding community-level responses to temperature variability across different phyla, size spectra, trophic levels and functional groups is therefore of critical importance.

Marine communities are particularly useful models for global change ecology studies because (i) they are often extremely diverse and comprise highly divergent taxa, from microbes to metazoans; (ii) they often comprise taxa that are short-lived and fast growing and thereby responsive to experimentation; and (iii) they are dominated by ectotherms that are strongly influenced by their surrounding environment and thermal variability. In contrast to both terrestrial [20,21] and freshwater [22,23] ecosystems, however, there have been very few field-based controlled manipulations of temperature in the marine environment. The vast majority of existing knowledge stems from laboratory-based microcosms/mesocosms, which typically focus on one or a few target species in isolation and are generally subjected to single uniform stressor treatments (e.g. constant temperature) that do not mimic natural variability [8].

We developed a novel experimental tool to conduct *in situ* temperature manipulations within natural marine habitats that support complex communities and diverse species pools. We tested the following hypotheses: (i) that increased temperature will alter patterns of diversity and community structure across multiple diverse biological groupings (i.e. from unicellular microorganisms through to metazoans), and (ii) that the pervasive community-level effects of increased temperature will be detectable at coarse taxonomic resolutions.

## 2. Methods

### (a) Experimental approach and design

Seawater temperature was elevated *in situ* with a heated settlement panel system (HSPS). Briefly, the HSPS comprised three sets of 10 replicate panels (individual panel dimensions: 15 × 15 cm) mounted onto a fibreglass lattice frame (see electronic supplementary material, figure S1). The first set of 10 panels were each heated with an electrical heat pad fixed to the underside of a stainless steel plate, and controlled by a series of microprocessors linked to a temperature sensor embedded onto each panel surface. The second set of 10 panels were heated in a similar way and a third set of 10 panels served as experimental controls held at ambient seawater temperature (full details and additional data are presented in the electronic supplementary material: methods statement and figures S1–S3). The desired temperature increase for each treatment (ambient temperature, +3°C and +5°C) was programmed into a shore-based control unit (see electronic supplementary material, figure S1), which maintained constant communications to the microprocessor units to precisely control temperatures over the panels.

**Figure 1.**
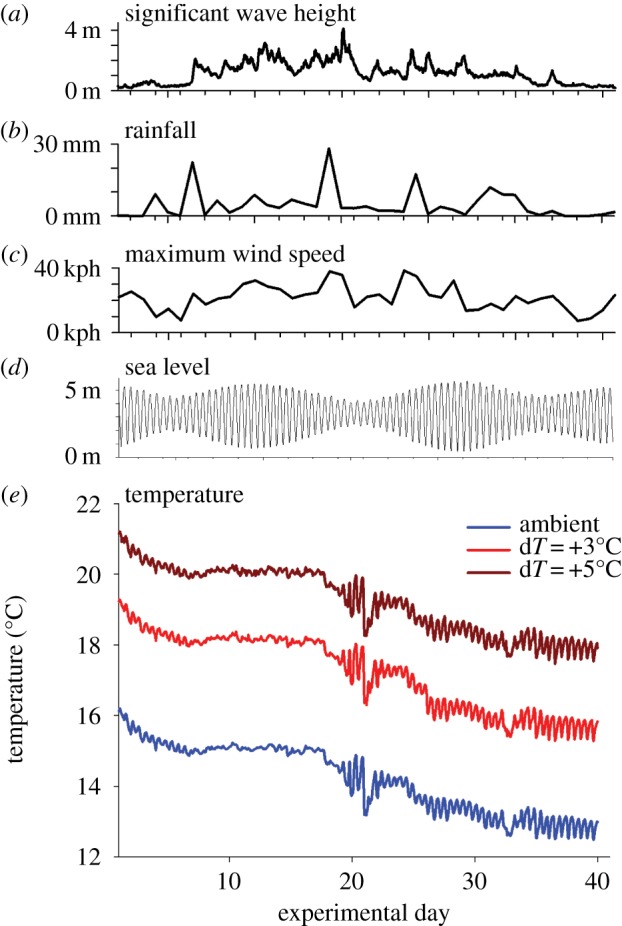
Local environmental conditions and experimental temperatures for each warming treatment. Top plots show local conditions of (*a*) wave height (adjacent open coast), (*b*) rainfall, (*c*) wind speed and (*d*) sea level during the experiment. Bottom plot (*e*) shows average temperature (*n* = 10 plates) for each treatment (ambient, +3°C and +5°C) over the 40-day experiment, which ran from October to November 2013. (Online version in colour.)

The field experiment was conducted within a marina in Plymouth, UK, between 10 October and 18 November 2013. An experimental settling surface (polyester fabric with a 700 µm pore size) was mounted onto each panel to provide a surface for colonization by marine organisms. A glass microscope slide was also secured to each panel, ensuring that it was mounted against the settling surface and within the heated boundary layer. The HSPS was deployed horizontally, suspended from a pontoon at approximately 2 m depth, with the plate surfaces facing downwards. Microbial and metazoan communities were sampled after 18 and 40 days, respectively, which allowed adequate time for biofilms and sessile invertebrates to cover more than 50% of all microscope slides/panel surfaces. Previous work has shown that assemblages of bacteria and protists associated with submerged substrata in marine habitats reach maturity within this timeframe [24–26], while approximately 1 month is sufficient time to allow for the colonization and development of sessile invertebrate assemblages [27,28]. Experimental trials indicated that the depth of the boundary layer of warmed seawater ranged from approximately 2 to more than 8 mm from the panel surface, depending on flow conditions (electronic supplementary material, figure S2). As such, the depth of warming treatment would be greater than that of the developing biofilm and of early-stage sessile invertebrate species, which were therefore continuously subjected to the temperature treatments. The HSPS facilitated precise control of *in situ* seawater temperature within a highly-dynamic coastal marine habitat, with the desired warming treatments of +3°C and +5°C above ambient sea temperature maintained for 40 days (figure 1*a–e*). Temperatures over the experimental panels precisely tracked natural variability in ambient sea temperature, which was of the order of 3°C throughout the experiment and was related to tidal cycles and storm events (figure 1). Experimental temperatures were highly correlated with ambient sea temperature (*r*^2^ > 0.998 for both treatments, see electronic supplementary material, figure S4) and the target treatments were maintained within ±0.2°C throughout the experiment (see electronic supplementary material, figure S4).

### (b) Quantifying diversity, abundance and community structure

After 18 days of *in situ* manipulation, microbial diversity was assessed by biofilm DNA extraction and bacterial 16S and eukaryote 18S rRNA gene high-throughput sequencing. Biofilms were removed from five replicate microscope slides (selected at random from each treatment) using a sterile razor blade before DNA was extracted and stored at −20°C. Standard PCR and sequencing techniques were employed (see electronic supplementary material; methods) and QIIME [29] was used to process the sequence data as described in detail previously [30,31]. In summary, quality filters were used to remove short (less than 200 bp) and low-quality reads (average phred score less than 25). Chimeric sequences were identified against the Greengenes reference database (release 13_5) [32] and removed. Operational taxonomic units (OTUs) were defined at 97% similarity and classified against the Greengenes reference database. Sequences are available from the European Nucleotide Archive (PRJEB18120). The total abundance of bacterial and microbial eukaryotes (primarily protists) after 18 days of immersion was quantified using quantitative PCR of bacterial 16S and eukaryote 18S rRNA genes, using well-established protocols (see electronic supplementary material; methods). Warming treatments were maintained for 40 days in total, after which time the settling surfaces were removed from the HSPS and preserved in ethanol for subsequent identification and quantification of macroscopic metazoans. The inner 13 × 13 cm of each surface was analysed by overlaying a transparency with grid markings to form 1 mm^2^ subunits. The metazoan taxa within each 1 × 1 mm grid-square were identified to the lowest taxonomic level possible (to species for 75% of all taxa and to genus for the remaining 25%) and the total number of subunits within which each taxon occurred was summed for each sample to quantify areal cover. The total abundance (i.e. number of individuals or distinct colonies) of each taxon was also recorded.

### (c) Statistical analysis

Community-level response patterns were analysed using multivariate statistics. The relative abundances of the OTUs determined by molecular methods and the abundance/cover of taxa identified with traditional taxonomic approaches were analysed separately but in a directly comparable way. Abundance data were initially square-root transformed to down-weight the influence of highly abundant taxa before constructing Bray–Curtis similarity matrices. PERMANOVA [33] was used to test for differences between the treatments (more than 999 unique unrestricted permutations) and where significant effects were detected (at *p* < 0.05) pairwise tests were conducted to determine which treatments differed from one another. Univariate community-level metrics were examined with permutational ANOVA. Similarity matrices based on Euclidean distances between untransformed abundance and richness values were constructed prior to conducting 999 unique unrestricted permutations to test for differences between treatments. Where significant differences in multivariate community structure between warming treatments were detected (at *p* < 0.05), SIMPER analysis was conducted to determine which OTUs/taxa contributed most to the observed differences. All statistical procedures were conducted using PRIMER v7 software [34] with the PERMANOVA add-on [35].

## 3. Results

**Figure 2.**
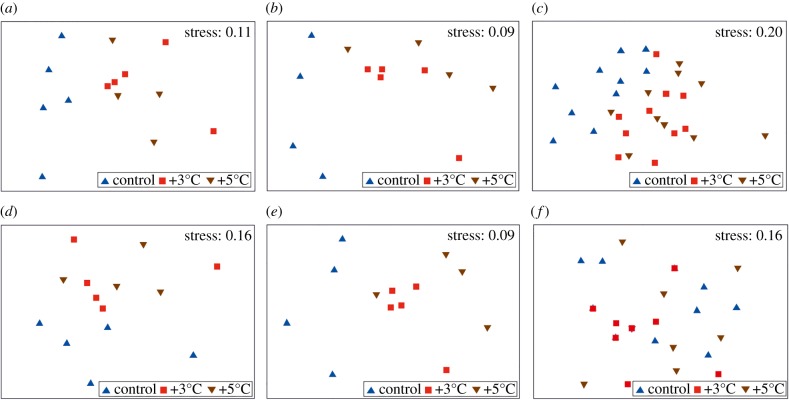
The effect of experimental warming on multivariate structure of multi-kingdom communities. Multidimensional scaling plots depicting communities of (*a*) bacteria and (*b*) protists after 18 days and communities of (*c*) metazoans (sessile invertebrates) after 40 days under each experimental treatment. Ordinations are based on a Bray–Curtis similarity matrix generated from square-root-transformed abundance data. Also shown are multidimensional scaling plots generated from presence/absence data describing communities of (*d*) bacteria and (*e*) protists after 18 days and communities of (*f*) metazoans (sessile invertebrates) after 40 days under each experimental treatment. (Online version in colour.)

In total, 2465 and 388 distinct OTUs were determined for bacteria and protists, respectively, and 12 distinct metazoan taxa (mostly bryozoa and ascidia) were identified. Multivariate analyses showed that communities comprising bacteria, protists and metazoans responded analogously to warming, in that communities held under ambient conditions were distinct from those that had developed under the warming treatments (figure 2*a*–*c*). PERMANOVA [35] tests showed significant differences between the experimental treatments, with the control communities being statistically distinct from those held under +3°C and +5°C (table 1). This response pattern was highly consistent between the major groupings (i.e. bacteria, protists and metazoans). Multivariate patterns based on presence/absence data were also examined, to assess the degree of community turnover between temperature treatments and community types. Variability patterns based on presence/absence data were similar to those based on square-root-transformed data for both bacteria and protists (figure 2*d*,*e*), as communities developed under warmed conditions were significantly different from those held at ambient temperature (table 1). For metazoans, however, multivariate patterns based on presence/absence data did not indicate separation between temperature treatments (figure 2*f*) and PERMANOVA tests did not detect significant between-treatment variability (table 1). For all comparisons, the PERMDISP routine indicated that within-treatment variability did not differ between treatments (*p* > 0.1 in all cases), suggesting that multivariate community structure, rather than multivariate dispersion (i.e. variability between plates), was impacted by increased seawater temperature.

**Table 1.** Permutational analyses to test for community-level differences between experimental warming treatments. (*a*) Variability in multivariate community structure was examined with PERMANOVA. Taxa abundances were square-root transformed prior to constructing a Bray–Curtis similarity matrix. Analyses were based on 999 unique unrestricted permutations. (*b*) Results of PERMANOVA tests to examine variability in multivariate community structure as described by presence/absence data. (*c*) Variability in community-level metrics was examined with univariate permutational analysis. Similarity matrices based on Euclidean distances between untransformed abundance and richness values were constructed prior to conducting 999 unique unrestricted permutations. Where significant differences were detected (at *p* < 0.05, given in italics) pairwise comparisons were conducted to determine which treatment levels differed from one another. The degrees of freedom associated with each test are shown in subscripted parentheses.

response variable	SS	MS	*F*	*p*	pairwise tests
(*a*) multivariate community structure (SQRT)					
bacteria _(2,11)_	2913	1456	1.606	*0.001*	C ≠ Δ3&Δ5, Δ3 = Δ5
protists _(2,10)_	7355	3677	1.673	*0.007*	C ≠ Δ3&Δ5, Δ3 = Δ
metazoans _(2,27)_	4621	2310	4.259	*0.001*	C ≠ Δ3&Δ5, Δ3 = Δ5
(*b*) multivariate community structure (P/A)					
bacteria _(2,11)_	2644	1322	1.200	*0.013*	C ≠ Δ3&Δ5, Δ3 = Δ5
protists _(2,10)_	7245	3622	1.736	*0.003*	C ≠ Δ3&Δ5, Δ3 = Δ
metazoans _(2,27)_	832	416	1.340	0.301	n.a.
(*c*) univariate community metrics					
bacteria abundance _(2,10)_	7.26 × 10^13^	3.63 × 10^13^	4.736	*0.036*	C < Δ3 = Δ5
eukaryote abundance _(2,10)_	1.46 × 10^12^	7.33 × 10^11^	1.076	0.375	n.a.
metazoan abundance _(2,27)_	10 120	5060	3.640	*0.004*	C < Δ3 = Δ5
bacteria richness _(2,11)_	33 676	16 838	2.883	0.070	n.a.
protist richness _(2,10)_	3452	1726	4.900	*0.042*	C < Δ3 = Δ5
metazoan richness _(2,27)_	5.60	2.80	2.634	0.114	n.a.

**Figure 3.**
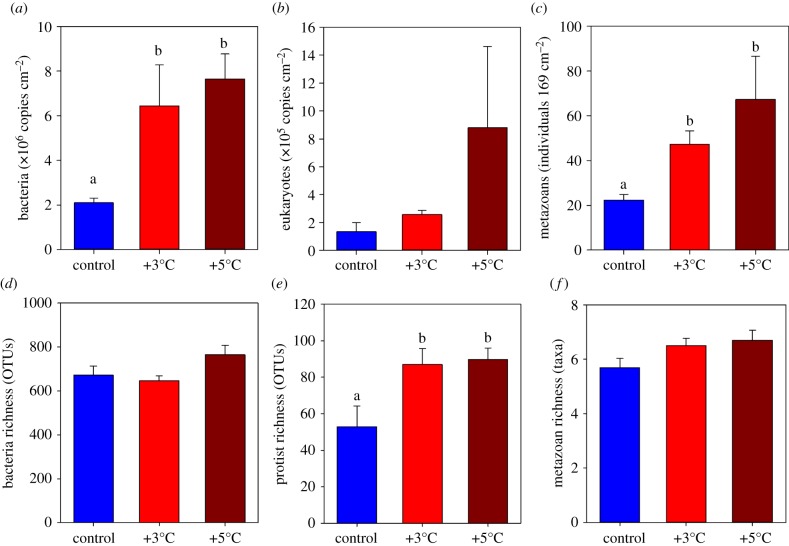
Effects of experimental warming on the abundance and richness of multi-kingdom communities. Mean (±s.e.) total abundance of communities of (*a*) bacteria and (*b*) protists after 18 days and communities of (*c*) metazoans (sessile invertebrates) after 40 days under each experimental treatment. Mean (±s.e.) total richness of communities of (*d*) bacteria and (*e*) protists after 18 days and communities of (*f*) metazoans (sessile invertebrates) also shown. Lower-case letters indicate differences between groups (pairwise tests) where a significant difference was detected between treatments (at *p* < 0.05, determined by univariate permutational ANOVA). (Online version in colour.)

For all community types, the total abundance of organisms increased in response to warming (figure 3*a*–*c*) with, on average, communities comprising three to six times as many organisms/marker genes in the +5°C treatments compared with the controls. Variability between temperature treatments was significant for the bacteria and metazoan communities, but not for the microbial eukaryotes owing to high within-treatment variability (figure 3, table 1). For bacteria and metazoan communities, the number of organisms recorded in the +3°C and +5°C samples was significantly higher than the samples held at ambient temperature (table 1). A similar pattern was observed for metazoan communities when structure was determined by the areal cover of sessile invertebrates (rather than the abundance); the warmed communities were more abundant and structurally distinct compared with the control communities (see electronic supplementary material, figure S5, table S1).

Patterns of community richness, as determined by the number of bacterial OTUs, eukaryote OTUs or the number of invertebrate taxa, also showed a similar response in that communities held at +5°C had consistently greater richness values than those held at ambient temperature (figure 3*d*–*f*). While this trend was apparent for all the groups, statistically significant differences between treatments were observed only for protists (table 1). The recorded number of OTUs/taxa that were unique to particular treatments did not vary in any consistent way between treatments or kingdoms (electronic supplementary material, figure S6).

With regards to the responses of individual OTUs/taxa to warming, a SIMPER analysis was conducted to determine the variables that were the principal contributors to the observed dissimilarity in community structure between the treatments. For the bacterial communities, *Rhodobacteria* were consistently important discriminators between the control and the warmed communities (see electronic supplementary material, table S2). For the protist communities, OTUs closely related to the diatom *Melosira varians* and the dinoflagellate *Paradinium poucheti* were less abundant at higher temperatures, whereas several other taxa (e.g. *Takayama* cf. *pulchellum*, *Telonema* sp.) showed the opposite pattern and were important discriminators between treatments (see electronic supplementary material; table S2). The metazoan communities were dominated by colonial and solitary ascidians and cheilostome bryozoans, which were all more abundant on warmed panels compared with controls (see electronic supplementary material, table S2). The UK-native ascidians *Diplosoma listerianum* and *Ciona intestinalis,* and the non-native ascidian *Corella eumyota*, were major contributors to the observed dissimilarity between the communities, and were all more abundant on panels held at +3°C and +5°C compared with those held at ambient temperature (see electronic supplementary material, table S2).

**Figure 4.**
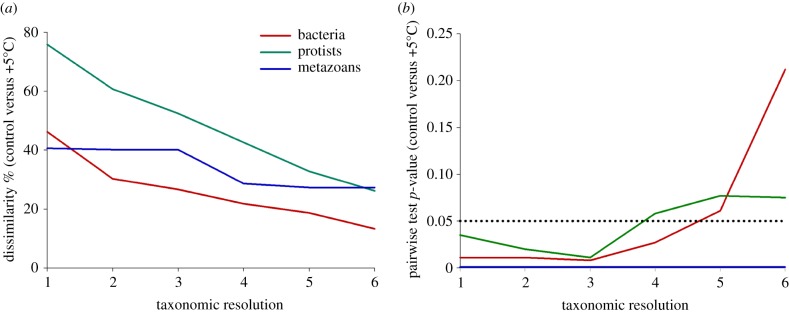
The influence of taxonomic resolution on the observed differences between communities held at ambient and increased temperatures. (*a*) The observed dissimilarity (%) between communities held at ambient temperature and those at +5°C at different taxonomic resolutions for each community type. (*b*) The significance level (*p*-value) derived from pairwise tests to detect differences in communities held at ambient temperature and those at +5°C at different taxonomic resolutions for each community type. The taxonomic resolution represents the decrease in precision from 1 (species/OTU) to 6 (phylum, level 5). (Online version in colour.)

Community-level responses to increased temperature were examined across a range of taxonomic resolutions by consolidating species/OTU-level data according to their taxonomic position (see electronic supplementary material, table S3, for taxonomic groupings used for protists). For bacteria, partitioning in multivariate community structure by treatment was evident through to aggregation to order level (see electronic supplementary material, figure S7). Order-level communities held at ambient temperature and at +5°C were significantly different, with overall dissimilarity at 21.8% (figure 4 and electronic supplementary material, table S4). For protists, clear and statistically significant partitioning in community structure between treatments was observed up to the third level of taxonomic aggregation (see electronic supplementary material, figure S7), after which communities became more similar and pairwise comparisons were non-significant (figure 4, see electronic supplementary material, table S4). Even at the coarsest level of taxonomic resolution, overall dissimilarity between protist communities held at ambient temperature and at +5°C was 26.1%. Dissimilarity between metazoan communities held under different temperature treatments was more consistent throughout aggregation to coarser taxonomic levels, and clear partitioning between treatments was observed at the phylum level (see electronic supplementary material, figure S7). Statistically, phylum-level metazoan communities held at ambient temperature and at +5°C were significantly different, with overall dissimilarity at 27.3% (figure 4 and electronic supplementary material, table S4).

## 4. Discussion

Our study has experimentally shown the overarching importance of seawater temperature in driving the development and structure of marine communities comprising a diversity of taxa, from microbes to metazoans. While the importance of temperature in determining the biology of marine ectotherms has been known for decades [14,36], only recently have technological advances allowed for experimental work on the underlying role of temperature variability in structuring natural communities. This is particularly true for marine communities, as controlling seawater temperature *in situ* is logistically challenging and, consequentially, very few studies have manipulated temperature within marine habitats (but see [28,37]), and no previous experiments have achieved such precise temperature control. Here, seawater warming resulted in significant shifts in community structure, which were similar in magnitude and direction across bacteria, protists and metazoans. Moreover, ecological responses to warming were evident even at coarse taxonomic scales, indicating that fundamental and pervasive shifts in community organization occurred (i.e. changes in the abundances of higher-order taxa rather than replacements of closely-related species), which would probably alter ecological functioning as well as structure. Our results show that temperature is a key driver of community development and succession in marine habitats, which supports and elaborates on previous work focusing on individual community-types such as bacteria-dominated aquatic biofilms [38] and marine assemblages comprising sessile invertebrates [39], which also highlighted temperature variability as a critical process (but see [27] for an example of limited temperature effects on metazoan communities).

Our most striking finding was the consistency of responses to warming across highly divergent communities, from bacteria to protists to metazoans, which operate at vastly different spatial and temporal scales and comprise fundamentally distinct organisms and taxa. Shifts in community structure were primarily driven by increased abundances at higher temperatures, as many key discriminatory taxa responded positively to warming and total community abundance generally increased with temperature across all community types. In addition, total community richness tended to be greater at the warmest treatment compared with the ambient controls. As temperature controls processes acting across biological scales, from genes [16] to ecosystems [13], increased temperature can lead to improved fitness and enhanced ecological performance as the thermal optimum of a species or population is reached, before declining rapidly as thermal thresholds are exceeded [15,40]. It is likely, however, that the maximum temperatures experienced during this experiment (approx. 19–21°C) fell below thermal thresholds for most taxa, many of which are widely distributed and occur in warmer waters [41–43], and experience temperature maxima of a similar magnitude during the summer months within the study region [44,45]. As such, many taxa are likely to have responded positively to higher temperatures, and exhibited faster growth and development on warmed surfaces.

Previous work on aquatic biofilms developed under different thermal regimes has shown that modest temperature increases of the order of 2–5°C can have considerable effects on the development, structure and functioning of communities comprising bacteria and other single-celled organisms [3,38,46–48]. Several studies have shown positive effects of warming, in that biofilms develop more quickly at elevated temperatures [3,47,49], while other studies have documented changes in the relative abundances of bacteria and protists [38,46,50]. With regards to the metazoans, it is well established that post-settlement and early development mortality, through disturbance or predation, is particularly high for many sessile invertebrates [51,52] and, as such, faster growth and maturation under warmer conditions may have reduced mortality rates and resulted in the higher abundances and spatial coverage of colonies and individuals observed at higher temperatures. If the experiment had been conducted during periods of maximum ambient temperatures (i.e. midsummer), or if trophic resources had become limiting, it is possible that increased temperature would have become stressful as thermal thresholds for some taxa were exceeded and, consequently, different population- and community-level responses would have been observed. Overall, our findings indicate that the rate of development of microbial biofilms and macroscopic sessile invertebrate communities is greater under warmed conditions, at least during periods when higher temperatures do not exceed annual maxima. Further work on the seasonality of community-level responses to warming is needed.

As well as a general increase in the abundances of bacteria and protists with warming, we recorded significant shifts in community composition (i.e. presence/absence data) between the temperature treatments, suggesting a high rate of turnover for these taxon-rich communities. Bacterial communities were particularly diverse and the number of unique taxa associated with each treatment was high (i.e. 250–400 OTUs); replacements of species in response to warming suggests some degree of selectivity or adaptation to temperature at the community-level. For the metazoans, however, there was no evidence of species turnover as multivariate communities based on presence/absence data did not differ between temperature treatments. Rather, shifts in metazoan community structure were related to changes in abundance rather than turnover of species or higher-order taxa. The higher richness of bacteria and protist communities and the greater diversity of the available species pool compared with metazoans may suggest that species turnover, and perhaps functional redundancy, in response to warming is comparatively limited for metazoans.

The rate and trajectory of microbial biofilm development is likely to have mediated, to some degree, the development of macroscopic metazoan communities, as settlement of sessile invertebrates is known to be influenced by biofilm community structure [53,54]. For example, recent experimental work has shown that seawater warming of the order of 2–6°C above ambient temperature can alter the structure of developing biofilm communities, which in turn may positively affect the settlement success of a coral reef sponge [55]. As such, the higher abundances of metazoans we observed under warmer conditions may have been caused, to some extent, by differences in the structure of recipient biofilms at each experimental temperature.

The most effective and reliable way of determining causation is through controlled experimentation [56], but manipulating temperature in natural marine environments is challenging. Here, the state-of-the-art HSPS facilitated precise temperature control to show experimentally that seawater temperature is a fundamental driver of community development and succession in marine environments across diverse taxonomic groupings. However, as with other experimental approaches, the HSPS does have limitations and the current study has certain caveats that should be considered when interpreting our results. First, the HSPS does not subject all components of populations and communities to increased temperature, and cannot test for warming effects on dispersal stages in the water column nor on highly mobile species such as mesograzers. It is likely that continued ocean warming will influence both dispersal capacity [57] and trophic interactions [58], and these types of ecological response warrant further research through other experimental approaches. Second, the HSPS approach is only appropriate for examining warming responses within a specific ecological window, as it can only be used for short-term manipulations and on early successional-stage sessile assemblages. Third, as previously stated, the current study reports on an experiment conducted in autumn and, as such, seasonal variability in responses to warming, carry-over effects between seasons and the importance of climatological history were not explored here and require further work. Even so, the HSPS does facilitate experimentation within complex habitats that are subjected to natural environmental variability and support representative species pools. In doing so, many of the issues of artificiality associated with even the most sophisticated mesocosm systems are largely removed and novel insights into the effects of temperature variability on whole, naturally-assembled communities can be gained.

While other anthropogenic stressors, such as ocean acidification [59] and eutrophication [60], are also important in determining ecological pattern, our study demonstrates that increased temperature is a major driver of change at both the population- and community-level. Warming events (i.e. ‘marine heatwaves’, see [61]) similar in magnitude and duration to our experimental treatments occur naturally in the oceans and may increase in severity as a consequence of anthropogenic climate change [61,62]. As such, current and predicted future changes in seawater temperature variability may drive fundamental and universal shifts in ecosystem structure with largely unknown impacts on ecosystem functioning.

## Supplementary Material

Additional methods and data relating to the experimental system and ecological responses

## Supplementary Material

Raw data

## Data accessibility

Raw data are available as electronic supplementary material.
