# Supplementary material for: Community responses to seawater warming are conserved across diverse biological groupings and taxonomic resolutions
Source: Proc Biol Sci. 2017 Sep 6;284(1862):20170534. doi: 10.1098/rspb.2017.0534 (PMC5597821; doi:10.1098/rspb.2017.0534)
Supplement: Additional methods and data relating to the experimental system and ecological responses [file rspb20170534supp1.docx]

**SUPPLEMENTARY INFORMATION**

Community-level responses to warming are conserved across diverse biological groupings and taxonomic resolutions

Dan A, Smale^1^, Joe D. Taylor^1,2^, Steve H. Coombs^1^, Gerald Moore^3^, and Michael Cunliffe^1,4*^

^1^Marine Biological Association of the United Kingdom, The Laboratory, Citadel Hill, Plymouth, PL1 2PB, United Kingdom

^2^Department of Biology, University of York, Wentworth Way, York, YO10 5DD, United Kingdom

^3^Bio-Optika, Gunnislake, PL18 9NQ, United Kingdom

^4^Marine Biology and Ecology Research Centre, School of Biological and Marine Sciences, Plymouth University, Drake Circus, Plymouth, United Kingdom.

**Corresponding author:** Dan Smale

Email: [dansma@mba.ac.uk](mailto:dansma@mba.ac.uk); Phone: +44 (0)1752 426489; Fax: +44(0)1752 633102

**Additional methods information**

**1. The Heated Settlement Panel System (HSPS)**

The HSPS comprised 3 sets of 10 replicate panels. The first set of panels formed an experimental treatment (*n* = 10) and were controlled by a series of microprocessors contained within a waterproof housing and mounted on the reverse face of the frame; a 50 m-length cable connected the housing to a shore-based control unit and power supply (Figure S1). The second set of panels represented another experimental treatment (*n* = 10) and were controlled and powered by a separate control unit and power supply (Figure S1). A third set of panels (*n* = 10), which were identical in design and composition but without electronics components, served as experimental controls that were held at ambient seawater temperature. The panels were pseudo-randomly allocated a position on the frame to ensure treatments were interspersed across the HSPS.

Panels were 15 x 15 cm and were mounted >10 cm apart from one another. Each experimental panel within the HSPS comprised a stainless steel plate on the underside of which an electrical heat pad was fixed and potted into place using an epoxy sealing compound. A temperature sensor was embedded on the surface of each stainless steel plate and both the heat pad and the temperature sensor were wired back to the microprocessor unit. Two additional temperature sensors were mounted on the reverse of the frame and wired into each microprocessor unit to record ambient seawater temperature. The desired temperature increase for each treatment (ambient temperature, +3°C and +5°C) was programmed into the shore-based control unit, which maintained constant communications to the microprocessor unit to precisely control temperatures over the panels. The shore-based control unit also housed a data logging system with USB interface, a real-time display to show temperatures over each experimental panel, a transformer to reduce working voltage from 240v to 55v and various safety measures (Figure S1). Throughout the experiment, temperatures over each panel as well as ambient temperature were recorded and logged every minute.

Extensive laboratory trials in seawater tanks were performed prior to experimentation to characterise the temperature gradient from the heated steel plate, through the settling surface (see below) and into the boundary layer of overlying seawater under different flow conditions. The magnitude of warming decreased rapidly with increasing distance from the plate surface in all flow conditions. Under high flow conditions warming was still detectable at 3 mm from the plate surface (Figure S2). With no flow, elevated temperatures were detectable at least 9 mm from the plate surface and, as such, microbial biofilms and early-stage sessile invertebrate communities would be within the layer of heated seawater in field conditions. In addition, the electromagnetic field generated by the plates was measured in the laboratory, in order to determine whether differences in temperature between treatments could be confounded by differences in the strength of any associated electromagnetic field. The HSPS was held in seawater and the warming treatments were allowed to stabilise before quantifying the electromagnetic field strength over the surface of replicate plates within each treatment. Multiple readings were taken across the surface of each replicate plate (n =3 per treatment) and no significant differences in the strength of the electromagnetic field between treatments were detected (Figure S3).

The field experiment was conducted in Millbay Marina, Plymouth, UK (50°21'47.20"N, 04° 9'7.90"W) and ran between 10^th^ October and 18^th^ November 2013. The study site within Plymouth Sound is influenced by strong tidal currents and occasional freshwater intrusion events following heavy rainfall. Artificial substrata at the study site support rich communities of marine flora and fauna comprising both native and non-native species, many of which are also found on nearby natural substrata. An experimental settling surface (polyester fabric with a 700 µm pore size) was mounted onto each panel to provide a surface for attachment. A glass microscope slide was secured to each plate, ensuring that it was mounted against the settling surface and within the heated boundary layer. The HSPS was deployed horizontally, suspended from a pontoon at ~2 m depth, with the plate surfaces facing downwards. Microbial and metazoan communities were sampled after 18 and 40 days, respectively, as this allowed adequate time for biofilms and sessile invertebrates to cover >50% of all microscope slides/panel surfaces. For the microbial communities, 5 replicate microscope slides were selected at random from each treatment, removed, and returned to the laboratory in seawater and then frozen at -80°C for subsequent analysis. Warming treatments were maintained for 40 days, after which time the settling surfaces were removed from the HSPS and preserved in ethanol for subsequent identification and quantification of macroscopic metazoans.

**2. Quantifying microbial communities**

Biofilms were removed from the microscope slides using a sterile razor blade before DNA was extracted using the PowerSoil DNA isolation kit (MO-BIO Laboratories, USA) and stored at -20°C. Polymerase chain reaction (PCR) and sequencing was carried as previously described (Taylor et al. 2014, Taylor and Cunliffe 2015). In brief, the V4 variable region of the bacterial 16S rRNA gene was amplified using the PCR primers 515F and 806R (Caporaso et al. 2011), and the following PCR conditions: 94°C for 3 minutes, followed by 28 cycles of 94°C for 30 seconds, 53°C for 40 seconds and 72°C for 1 minute, and a final elongation step at 72°C for 5 minutes. The V9 variable region of the eukaryote 18S rRNA gene was amplified using the PCR primers 1391F and EukB (Amaral-Zettler et al. 2009) and the following PCR conditions: 94°C for 3 minutes, followed by 28 cycles of 94°C for 30 seconds, 53°C for 40 seconds and 72°C for 1 minute, and a final elongation step at 72°C for 5 minutes. Sequencing of amplified 16S rRNA and 18S rRNA genes was performed on an Ion Torrent PGM (Life technologies). QIIME (Caporaso et al. 2010) was used to process the sequence data as described in detail previously (Taylor et al. 2014, Taylor and Cunliffe 2015). In summary, quality filters were used to remove short (<200 bp) and low quality reads (average phred score <25). Chimeric sequences were identified against the Greengenes reference database (release 13_5) (DeSantis et al. 2006) and removed. Operational taxonomic units (OTUs) were defined at 97% similarity and classified against the Greengenes reference database. Sequences are available from the European Nucleotide Archive (PRJEB18120).

Bacteria Q-PCR was performed using primers BACT1369F and PROK1492R (Suzuki et al. 2000). Eukaryote QPCR was performed with primers EUK345F and EUK499R (Zhu et al. 2005). QPCR was carried out using the Sensi-FAST SYBR Q-PCR kit (Bioline) in 10µl reactions containing 5 μL of sensi-fast master mix, 0.25 μL of each primer (final concentration 0.4 μM), 1 μL of DNA template and 4 μL nuclease free water. A Qiagen Rotor Gene3000 (Qiagen) was used to perform the reactions. Cycling conditions were an initial denaturation of 94^°^C for 3 min, then 40 cycles of 94^°^C for 10s, annealing for 15s at 59^°^C for bacteria or 60°C for eukaryotes, elongation and acquisition of fluorescence data at 72^°^C for 20s. Standard curves for Q-PCR were constructed using known amounts of purified target template generated by PCR ampliﬁcation of the target gene from genomic DNA from either *Escherichia coli* or *Saccharomyces cerevisiae*.

**FIGURE S1.** The Heated Settlement Panel System (HSPS) used to conduct *in situ* temperature manipulations within a dynamic marine habitat. The approach used electrical heat pads embedded below stainless steel plates to elevate the temperature of the substratum and surrounding boundary layer of sea water. The HSPS comprised 3 x 10 replicate plates mounted onto a fibreglass lattice frame (c). Two sets of 10 plates were each held at 2 elevated temperatures and were controlled by a series of microprocessors contained within a waterproof housing and mounted on the reverse face of the frame (d). A third set of plates were unheated and acted as the controls. A 50 m-length cable connected the housed microprocessors to a shore-based control unit and power supply (a,b). The shore-based control unit housed a data logging system with USB interface, a real-time display to show temperatures over each experimental panel, a transformer to reduce working voltage from 240v to 55v and various safety measures (b,e). Throughout the experiment, temperatures over each panel as well as ambient temperature were recorded and logged every minute (e).


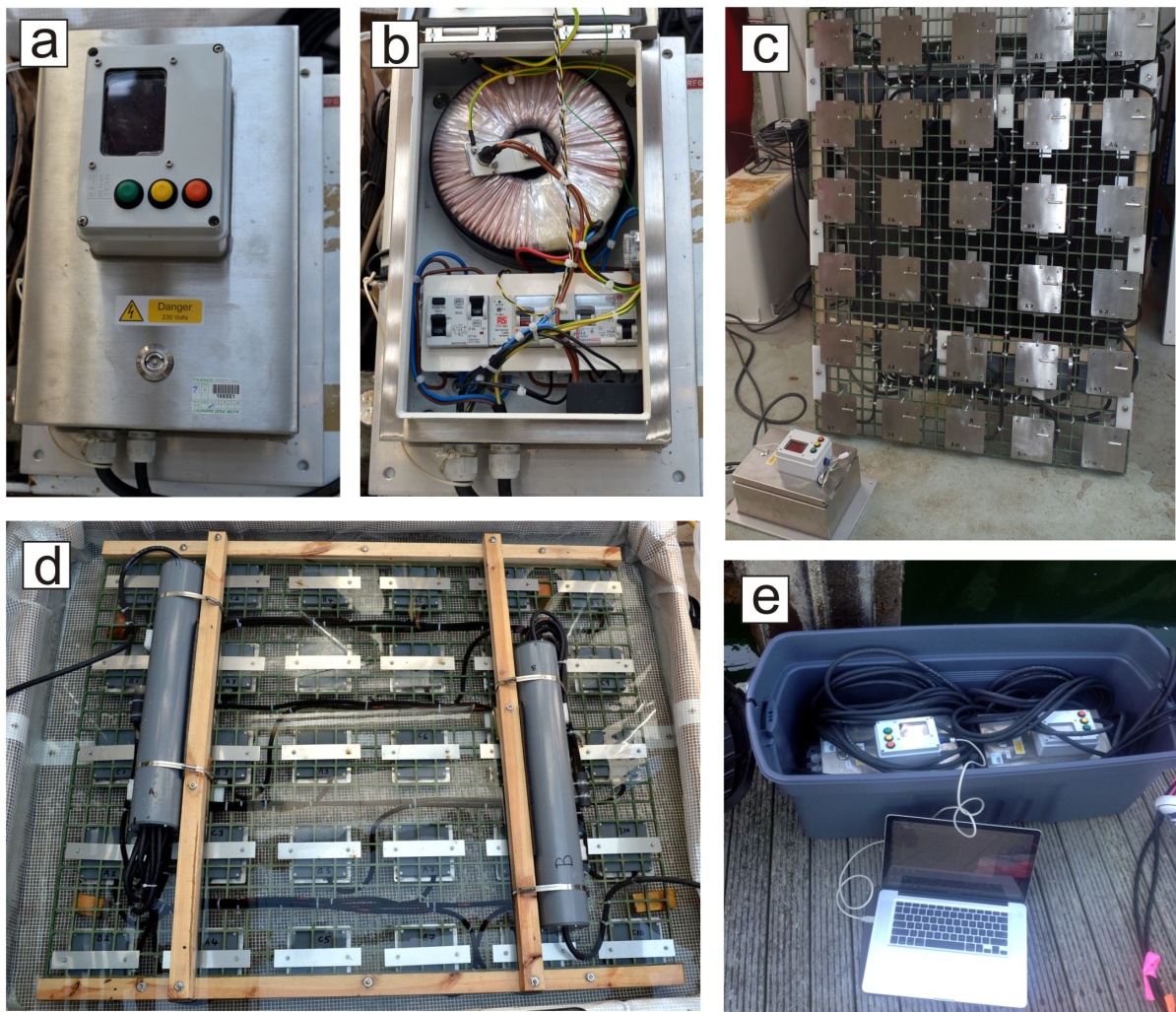


**FIGURE S2.** The extent of the warm water boundary layer under different flow conditions. The relationship between distance from the surface of the experimental plate and the observed increase in temperature for both the **(a)** +°5C and **(b)** +°3C and warming treatments, as determined by controlled experiments conducted under different flow conditions (medium flow: ~0.1 ms^-1^, high flow: ~0.3 ms^-1^).


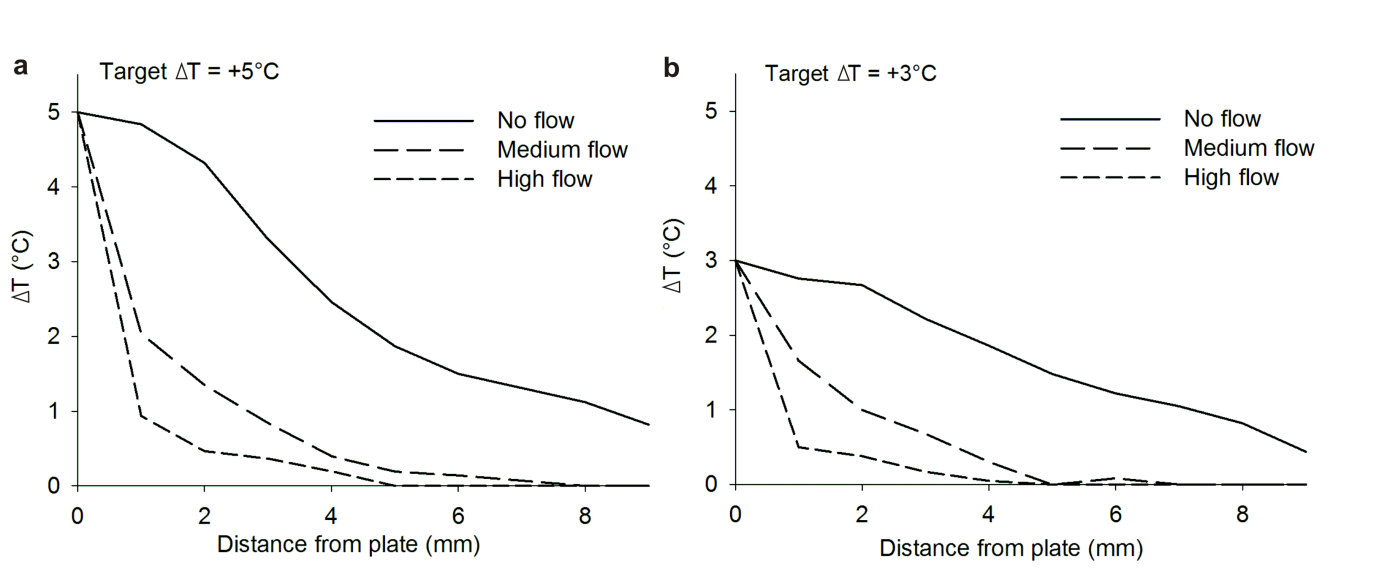


**FIGURE S3.** Mean strength of the electromagnetic field acting across plates of each experimental treatment. The electromagnetic field strength (i.e. magnetic vector, in µT) was measured in seawater and >24 readings were obtained across the plate surface. Mean values are calculated from 3 replicate plates per treatment.


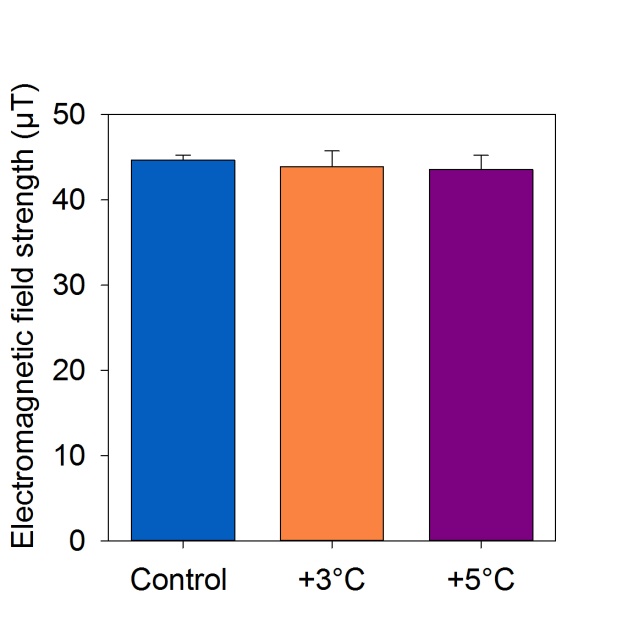


**FIGURE S4.** The performance of the experimental warming treatments. (a) The correlation between observed temperature on the treatment plates with ambient seawater temperature during the experiment. Histograms showing distributions of observed temperatures for the (b) +°3C and (c) +°5C warming treatments (938 hourly-averaged observations, n = 10 replicate plates per observation).





**FIGURE S5.** The structure of metazoan communities as quantified by areal cover measurements. (a) Total areal cover of sessile invertebrate communities held under each experimental treatment (lower case letters indicate significant differences) and (b) multivariate structure of metazoan communities for each experimental treatment (each centroid represents a single panel community).


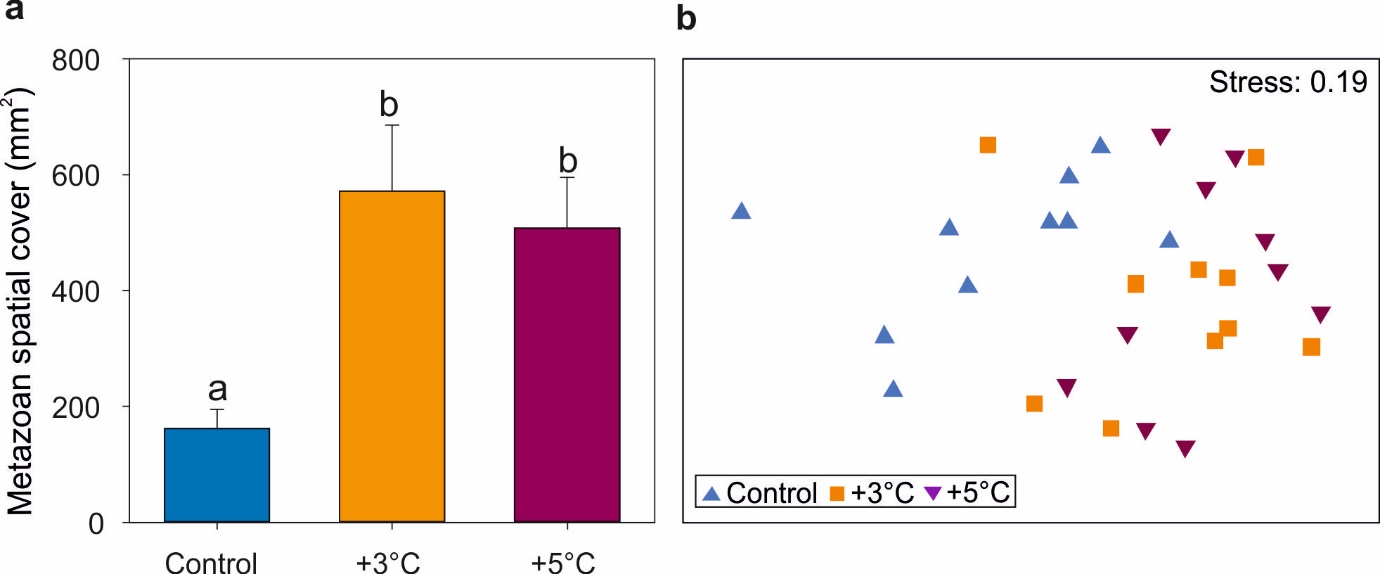


**FIGURE S6.** The number of OTUs/taxa unique to each experimental treatment for each community-type.


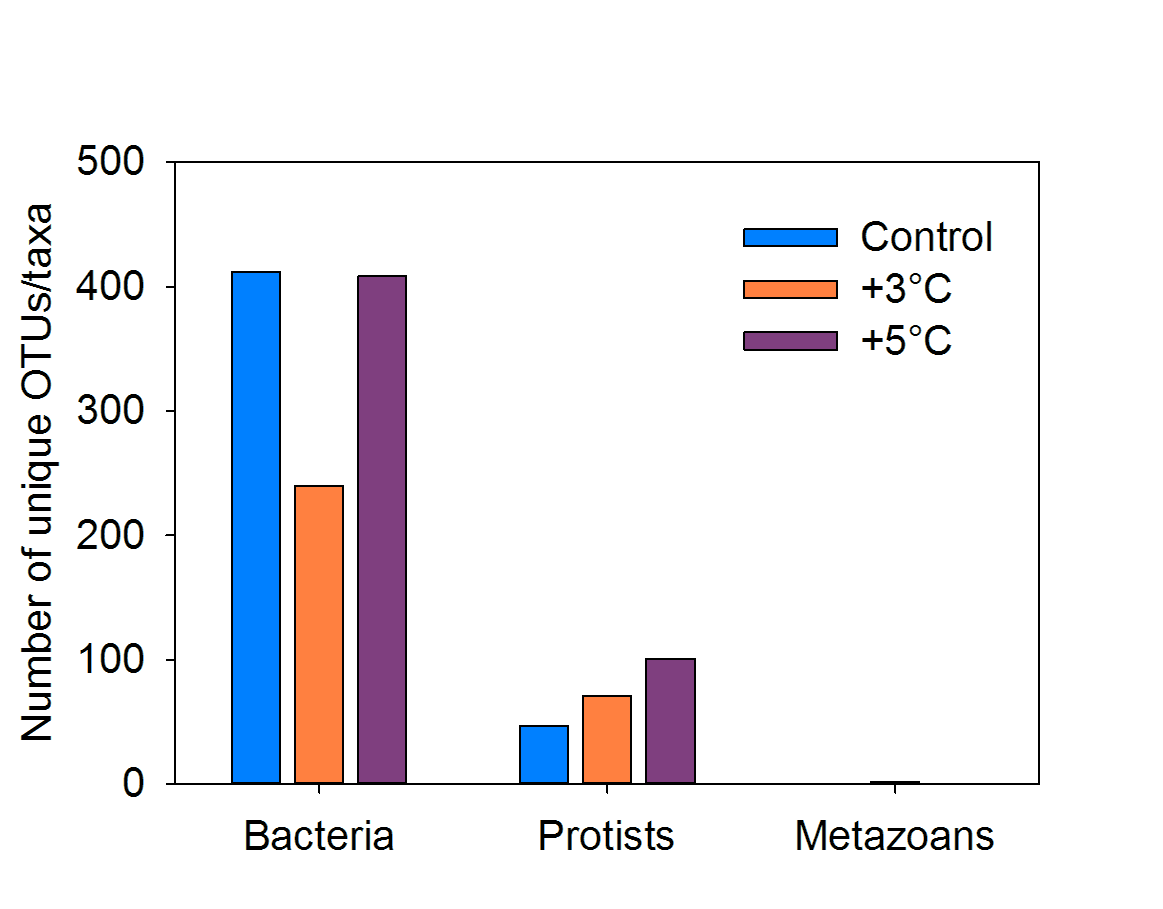


**FIGURE S7.** The effect of experimental warming on multivariate community structure at decreasing taxonomic resolutions**.** Multidimensional scaling (MDS) plots depicting communities of bacteria and protists after 18 days and communities of metazoans (sessile invertebrates) after 40 days under each experimental treatment. Ordination is based on a Bray-Curtis similarity matrix generated from square root transformed abundance data. Taxonomic resolutions shown for bacteria are (a) genus (b) family (c) order (d) class (e) phylum; for protists are (f) level 1 (g) level 2 (h) level 3 (i) level 4 (j) level 5; and for metazoans are (k) genus (l) family (m) order (n) class (o) phylum.


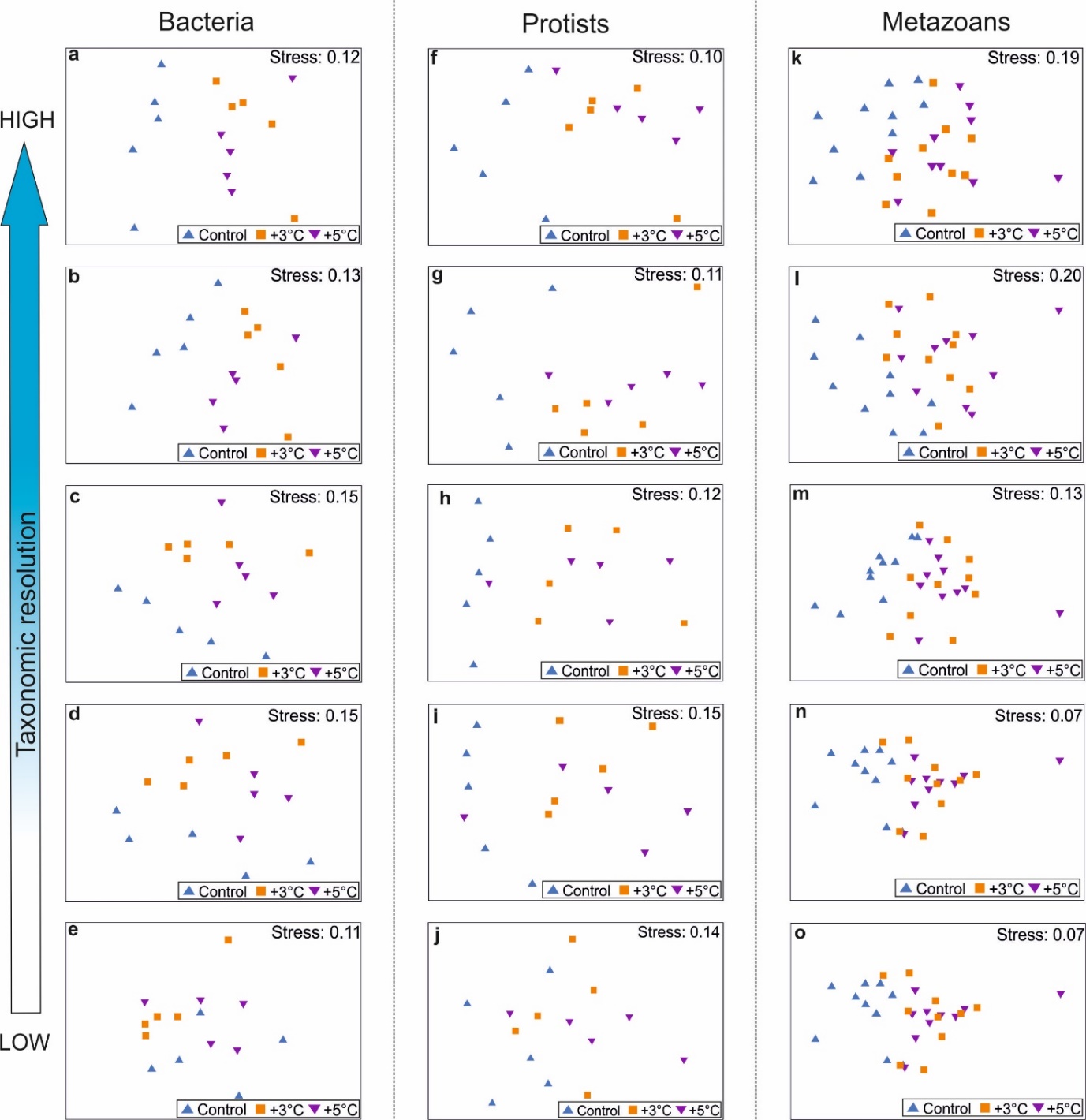


**Table S1.** Results of permutational analyses to test for differences between experimental warming treatments based on the areal cover of macroinvertebrates. Variability in total areal cover was examined with univariate permutational analysis. Similarity matrices based on Euclidean distances between untransformed areal cover values were constructed prior to conducting 999 unique unrestricted permutations. Variability in multivariate community structure as quantified by areal cover was examined with PERMANOVA. The abundances of response variables were square-root transformed prior to constructing a Bray-Curtis similarity matrix. Analyses were based on 999 unique unrestricted permutations.Where significant differences were detected (at P<0.05, shown in bold) pairwise comparisons were conducted to determine which treatment levels differed from one another. The degrees of freedom associated with each test are shown in subscripted parentheses. In both cases the PERMDISP routine did not detect differences in within-treatment variability.

**Response variable SS MS *F P* Pairwise** Metazoan total cover _(2,27)_ 9.72x10^5^ 4.86 x10^5^ 6.63 0.004 C<Δ3=Δ5

Metazoan community structure _(2,27)_ 5627 2813 3.50 0.002 C≠Δ3=Δ5

**Table S2.** SIMPER analysis to determine the relative contribution of individual OTUs/taxa to observed differences between warming treatments. For each comparison and OTU/taxon the average abundance in each treatment (‘Group 1 ab.’ and ‘Group 2 ab.’), average dissimilarity (‘Av. diss’), dissimilarity/standard deviation ratio (‘Diss./SD’), contribution to the observed difference (‘Contri.%’) and cumulative dissimilarity (‘Cum.%’) between treatments are shown. Top 5 contributors to the observed dissimilarity are shown for each comparison.

**Variable Group1 ab. Group2 ab. Av. diss. Diss./SD Contr. % Cum. %**

**a**: Bacteria communities

Control versus Δ3°C (46.3% dissimilarity)

*Thiomicrospira chilensis* 0.00 11.65 0.44 1.80 0.95 0.95

*Rhodobacteraceae* 1 1.08 11.86 0.41 1.22 0.89 1.84

*Rhodobacteraceae* 2 4.23 13.58 0.39 0.39 0.84 2.69

*Alteromonas* sp. 1.19 8.84 0.29 2.75 0.63 3.31

*Colwelliaceae* 1 0.20 7.38 0.27 2.06 0.59 3.90

Control versus Δ5°C (46.2% dissimilarity)

*Rhodobacteraceae* 2 4.23 14.95 0.38 1.59 0.83 0.83

*Thiomicrospira chilensis* 0.00 10.58 0.37 3.92 0.80 1.63

*Rhodobacteraceae* 3 31.92 22.16 0.37 1.65 0.80 2.43

*Rhodobacteraceae* 4 22.05 14.06 0.28 1.91 0.62 3.04

*Rhodobacteraceae* 1 1.08 8.22 0.25 1.27 0.54 3.59

**b**: Protist communities

Control versus Δ3°C (72.2% dissimilarity)

*Melosira varians*  8.41 3.75 2.09 1.59 2.90 2.90

*Paradinium poucheti* 5.78 2.55 1.42 1.74 1.96 4.86

*Takayama* cf. *pulchellum* 1.29 4.57 1.42 1.72 1.96 6.83

*Telonema* sp. 3.05 4.50 1.26 1.21 1.74 8.57

*Pentapharsodinium* sp. 0.91 4.03 1.21 1.48 1.68 10.25

Control versus Δ5°C (75.9% dissimilarity)

*Melosira varians*  8.41 3.30 2.04 1.74 2.69 2.69

*Paradinium poucheti* 5.78 3.34 1.23 2.00 1.62 4.31

Silicofilosea 1 2.57 0.00 0.95 0.95 1.25 5.56

*Apatococcus lobatus* 0.00 2.38 0.94 0.56 1.24 6.80

Kathablepharidae 1 0.00 2.48 0.92 1.59 1.22 8.02

**c**: Metazoan communities

Control versus Δ3°C (36.1% dissimilarity)

*Diplosoma listerianum* 2.97 3.95 7.41 1.43 20.56 20.56

*Corella eumyota* 2.11 3.60 6.27 2.15 17.38 37.97

*Ciona intestinalis* 1.17 2.40 6.24 1.53 17.31 55.25

*Tricellaria inopinata* 0.44 1.15 3.68 1.22 10.22 65.47

*Bugula fulva* 0.54 1.11 3.48 1.37 9.64 75.11

Control versus Δ5°C (40.6% dissimilarity)

*Corella eumyota* 2.11 4.25 7.90 2.25 19.44 19.44

*Diplosoma listerianum* 2.97 4.28 6.55 1.33 16.11 35.55

*Ciona intestinalis* 1.17 2.17 5.21 1.42 12.81 48.36

*Bugula fulva* 0.54 1.73 4.68 1.25 11.52 59.87

*Botryllus schlosseri* 0.64 1.54 4.05 1.52 9.95 69.82

**Table S3.** Hierarchical taxonomic groupings of microbial eukaryote (protist) operational taxonomic units from 18S rRNA gene libraries. Because protist OTU-based taxonomy is variable at higher levels we used the hierarchical taxonomic groupings from the Protist Ribosomal Reference database (PR2) as a scaffold. Groupings start at the second stage in the table below (i.e. Amoebozoa).

| Eukaryota;__Amoebozoa;__Conosa;__Dictyostelia;__Dictyostelium;__Dictyostelium_purpureum |
| --- |
| Eukaryota;__Amoebozoa;__Conosa;__Dictyostelia;__Dictyostelium;__Dictyostelium_vinaceofuscum |
| Eukaryota;__Amoebozoa;__Conosa;__Myxogastria;__Physarum;__Hyperamoeba |
| Eukaryota;__Amoebozoa;__Conosa;__Myxogastria;__Physarum;__Physarum_polycephalum |
| Eukaryota;__Amoebozoa;__Conosa;__Myxogastria;__Protophysarum;__Hyperamoeba |
| Eukaryota;__Amoebozoa;__Conosa;__Protosteliida;__CCW8;__Dinenympha |
| Eukaryota;__Amoebozoa;__Conosa;__Variosea;__Varipodida;__LEMD267 |
| Eukaryota;__Amoebozoa;__Conosa;__Variosea;__Varipodida;__soil_amoeba_AND16 |
| Eukaryota;__Amoebozoa;__Discosea;__Flabellinia;__Dactylopodida;__Korotnevella |
| Eukaryota;__Amoebozoa;__Discosea;__Flabellinia;__Dactylopodida;__Neoparamoeba |
| Eukaryota;__Amoebozoa;__Discosea;__Flabellinia;__Stygamoebida;__Vermistella |
| Eukaryota;__Amoebozoa;__Discosea;__Flabellinia;__Vannellida;__Platyamoeba |
| Eukaryota;__Amoebozoa;__Discosea;__Flabellinia;__Vannellida;__Protosteliopsis |
| Eukaryota;__Amoebozoa;__Discosea;__Flabellinia;__Vannellida;__Vannella |
| Eukaryota;__Amoebozoa;__Discosea;__Longamoebia;__Centramoebida;__Acanthamoeba |
| Eukaryota;__Amoebozoa;__Lobosa;__Tubulinea;__Echinamoebida;__Echinamoeba |
| Eukaryota;__Amoebozoa;__Lobosa;__Tubulinea;__Euamoebida;__BOLA868 |
| Eukaryota;__Amoebozoa;__Lobosa;__Tubulinea;__Euamoebida;__Hartmannella |
| Eukaryota;__Amoebozoa;__Lobosa;__Tubulinea;__Leptomyxida;__Elev-18S-666 |
| Eukaryota;__Amoebozoa;__Lobosa;__Tubulinea;__Leptomyxida;__Rhizamoeba |
| Eukaryota;__Archaeplastida;__Chloroplastida;__Chlorophyta;__Chlorodendrales;__Tetraselmis |
| Eukaryota;__Archaeplastida;__Chloroplastida;__Chlorophyta;__Chlorophyceae;__Desmodesmus |
| Eukaryota;__Archaeplastida;__Chloroplastida;__Chlorophyta;__Mamiellophyceae;__Mamiella |
| Eukaryota;__Archaeplastida;__Chloroplastida;__Chlorophyta;__Mamiellophyceae;__Micromonas |
| Eukaryota;__Archaeplastida;__Chloroplastida;__Chlorophyta;__Mamiellophyceae;__uncultured_marine_eukaryote |
| Eukaryota;__Archaeplastida;__Chloroplastida;__Chlorophyta;__Prasinophytae;__Pycnococcus |
| Eukaryota;__Archaeplastida;__Chloroplastida;__Chlorophyta;__Prasinophytae;__Pyramimonas |
| Eukaryota;__Archaeplastida;__Chloroplastida;__Chlorophyta;__Trebouxiophyceae;__AN_1-3 |
| Eukaryota;__Archaeplastida;__Chloroplastida;__Chlorophyta;__Trebouxiophyceae;__Apatococcus |
| Eukaryota;__Archaeplastida;__Chloroplastida;__Chlorophyta;__Trebouxiophyceae;__Botryococcus |
| Eukaryota;__Archaeplastida;__Chloroplastida;__Chlorophyta;__Trebouxiophyceae;__Chlorella |
| Eukaryota;__Archaeplastida;__Chloroplastida;__Chlorophyta;__Trebouxiophyceae;__Choricystis |
| Eukaryota;__Archaeplastida;__Chloroplastida;__Chlorophyta;__Trebouxiophyceae;__Leptosira |
| Eukaryota;__Archaeplastida;__Chloroplastida;__Chlorophyta;__Trebouxiophyceae;__Myrmecia |
| Eukaryota;__Archaeplastida;__Chloroplastida;__Chlorophyta;__Trebouxiophyceae;__Nannochloris |
| Eukaryota;__Archaeplastida;__Chloroplastida;__Chlorophyta;__Trebouxiophyceae;__Parachlorella |
| Eukaryota;__Centrohelida;__Heterophryidae;__Heterophrys;__Heterophrys_marina;Other |
| Eukaryota;__Centrohelida;__Heterophryidae;__Oxnerella;__Oxnerella_micra;Other |
| Eukaryota;__Centrohelida;__Heterophryidae;__uncultured_eukaryote;Other;Other |
| Eukaryota;__Centrohelida;__uncultured_eukaryote;Other;Other;Other |
| Eukaryota;__Cryptophyceae;__Cryptomonadales;__FV18-2G7;__Cryptophyceae_sp._CCMP2045;Other |
| Eukaryota;__Cryptophyceae;__Cryptomonadales;__FV18-2G7;__uncultured_marine_picoeukaryote;Other |
| Eukaryota;__Cryptophyceae;__Cryptomonadales;__Geminigera;__Geminigera_cryophila;Other |
| Eukaryota;__Cryptophyceae;__Cryptomonadales;__Hemiselmis;__Hemiselmis_cryptochromatica;Other |
| Eukaryota;__Cryptophyceae;__Cryptomonadales;__uncultured_marine_eukaryote;Other;Other |
| Eukaryota;__Cryptophyceae;__Goniomonas;__Goniomonas_sp._ATCC_50108;Other;Other |
| Eukaryota;__Cryptophyceae;__Goniomonas;__Goniomonas_sp._SH-3;Other;Other |
| Eukaryota;__Cryptophyceae;__P1-31;__uncultured_Proteomonas;Other;Other |
| Eukaryota;__Excavata;__Discoba;__Discicristata;__Euglenozoa;__Diplonemea |
| Eukaryota;__Excavata;__Discoba;__Discicristata;__Heterolobosea;__Tetramitia |
| Eukaryota;__Excavata;__Discoba;__Jakobida;__uncultured;__Scrupocellaria |
| Eukaryota;__Excavata;__Metamonada;__Preaxostyla;__Trimastix;__Trimastix_pyriformis_ATCC50562 |
| Eukaryota;__Haptophyta;__Prymnesiophyceae;__ESS2202;__uncultured_Emiliania;Other |
| Eukaryota;__Haptophyta;__Prymnesiophyceae;__ESS2202;__uncultured_haptophyte;Other |
| Eukaryota;__Haptophyta;__Prymnesiophyceae;__OLI16010;__eukaryote_clone_OLI11007;Other |
| Eukaryota;__Haptophyta;__Prymnesiophyceae;__Prymnesiales;__AI5F14RJ2A07;__uncultured_eukaryote |
| Eukaryota;__Haptophyta;__Prymnesiophyceae;__Prymnesiales;__Chrysochromulina;__Chrysochromulina_simplex |
| Eukaryota;__Haptophyta;__Prymnesiophyceae;__Prymnesiales;__Chrysochromulina;__Platychrysis |
| Eukaryota;__Haptophyta;__Prymnesiophyceae;__Prymnesiales;__Imantonia;__uncultured_marine_picoeukaryote |
| Eukaryota;__Haptophyta;__Prymnesiophyceae;__Prymnesiales;__OLI16029;__uncultured_marine_haptophyte |
| Eukaryota;__Haptophyta;__T58;__uncultured_marine_haptophyte;Other;Other |
| Eukaryota;__Incertae_Sedis;__Ancyromonadida;__Ancyromonas;__Ancyromonas_micra;Other |
| Eukaryota;__Incertae_Sedis;__Apusomonadidae;__Amastigomonas;__Amastigomonas_debruynei;Other |
| Eukaryota;__Incertae_Sedis;__Apusomonadidae;__Amastigomonas;__Amastigomonas_sp._2_Bamfield;Other |
| Eukaryota;__Incertae_Sedis;__Apusomonadidae;__Apusomonas;__Apusomonas_proboscidea;Other |
| Eukaryota;__Incertae_Sedis;__Palpitomonas;__marine_microheliozoan_TCS-2002;Other;Other |
| Eukaryota;__Incertae_Sedis;__Palpitomonas;__uncultured_dinoflagellate;Other;Other |
| Eukaryota;__Incertae_Sedis;__Telonema;__uncultured_eukaryote;Other;Other |
| Eukaryota;__Incertae_Sedis;__Telonema;__uncultured_marine_Telonemida;Other;Other |
| Eukaryota;__Kathablepharidae;__Katablepharis;__Katablepharis_remigera;Other;Other |
| Eukaryota;__Kathablepharidae;__Leucocryptos;__Leucocryptos_marina;Other;Other |
| Eukaryota;__Kathablepharidae;__Leucocryptos;__uncultured_marine_eukaryote;Other;Other |
| Eukaryota;__Kathablepharidae;__uncultured;__uncultured_katablepharidophyte;Other;Other |
| Eukaryota;__Kathablepharidae;__uncultured_katablepharidophyte;Other;Other;Other |
| Eukaryota;__Picozoa;__uncultured_Cyanophora;Other;Other;Other |
| Eukaryota;__Picozoa;__uncultured_marine_eukaryote;Other;Other;Other |
| Eukaryota;__Picozoa;__uncultured_rhodophyte;Other;Other;Other |
| Eukaryota;__SAR;__Alveolata;__Apicomplexa;__Conoidasida;__Coccidia |
| Eukaryota;__SAR;__Alveolata;__Apicomplexa;__Conoidasida;__Cryptosporida |
| Eukaryota;__SAR;__Alveolata;__Apicomplexa;__Novel_Apicomplexa_Class_1;__uncultured_eukaryote |
| Eukaryota;__SAR;__Alveolata;__Ciliophora;__Intramacronucleata;__Spirotrichea |
| Eukaryota;__SAR;__Alveolata;__Ciliophora;__Postciliodesmatophora;__Heterotrichea |
| Eukaryota;__SAR;__Alveolata;__Dinoflagellata;__Dinophyceae;__Dinophysiales |
| Eukaryota;__SAR;__Alveolata;__Dinoflagellata;__Dinophyceae;__Gymnodiniphycidae |
| Eukaryota;__SAR;__Alveolata;__Dinoflagellata;__Dinophyceae;__Peridiniphycidae |
| Eukaryota;__SAR;__Alveolata;__Dinoflagellata;__Dinophyceae;__Prorocentrales |
| Eukaryota;__SAR;__Alveolata;__Dinoflagellata;__Incertae_Sedis;__Blastodinium |
| Eukaryota;__SAR;__Alveolata;__Dinoflagellata;__Incertae_Sedis;__Haplozoon |
| Eukaryota;__SAR;__Alveolata;__Dinoflagellata;__Noctilucales;__Spatulodinium |
| Eukaryota;__SAR;__Alveolata;__NIF-4C10;__uncultured_marine_eukaryote;Other |
| Eukaryota;__SAR;__Alveolata;__Protalveolata;__Syndiniales;__Amoebophrya |
| Eukaryota;__SAR;__Alveolata;__Protalveolata;__Syndiniales;__Duboscquella |
| Eukaryota;__SAR;__Alveolata;__Protalveolata;__Syndiniales;__Syndiniales_Group_I |
| Eukaryota;__SAR;__Alveolata;__Protalveolata;__Syndiniales;__Syndiniales_Group_II |
| Eukaryota;__SAR;__Alveolata;__Protalveolata;__Syndiniales;__Syndiniales_Group_III |
| Eukaryota;__SAR;__Alveolata;__Protalveolata;__Syndiniales;__Syndiniales_Group_V |
| Eukaryota;__SAR;__Alveolata;__Protalveolata;__Syndiniales;__Syndinium |
| Eukaryota;__SAR;__Alveolata;__Protalveolata;__Syndiniales;__uncultured_eukaryote |
| Eukaryota;__SAR;__Rhizaria;__Cercozoa;__Cercomonadidae;__Cavernomonas |
| Eukaryota;__SAR;__Rhizaria;__Cercozoa;__Cercomonadidae;__Cercomonas |
| Eukaryota;__SAR;__Rhizaria;__Cercozoa;__Clathrulinidae;__Hedriocystis |
| Eukaryota;__SAR;__Rhizaria;__Cercozoa;__Endomyxa;__Ascetosporea |
| Eukaryota;__SAR;__Rhizaria;__Cercozoa;__Endomyxa;__Vampyrellidae |
| Eukaryota;__SAR;__Rhizaria;__Cercozoa;__Glissomonadida;__Heteromita |
| Eukaryota;__SAR;__Rhizaria;__Cercozoa;__Metromonadea;__Metopion |
| Eukaryota;__SAR;__Rhizaria;__Cercozoa;__Metromonadea;__Metromonas |
| Eukaryota;__SAR;__Rhizaria;__Cercozoa;__Metromonadea;__Micrometopion |
| Eukaryota;__SAR;__Rhizaria;__Cercozoa;__Novel_Clade_12;__uncultured_eukaryote |
| Eukaryota;__SAR;__Rhizaria;__Cercozoa;__Novel_Clade_2;__uncultured_cercozoan |
| Eukaryota;__SAR;__Rhizaria;__Cercozoa;__Novel_Clade_2;__uncultured_eukaryote |
| Eukaryota;__SAR;__Rhizaria;__Cercozoa;__Novel_Clade_Gran-4;__Exuviaella |
| Eukaryota;__SAR;__Rhizaria;__Cercozoa;__Novel_Clade_Gran-4;__uncultured_marine_eukaryote |
| Eukaryota;__SAR;__Rhizaria;__Cercozoa;__Silicofilosea;__Basal_Group_T |
| Eukaryota;__SAR;__Rhizaria;__Cercozoa;__Silicofilosea;__CCW10 |
| Eukaryota;__SAR;__Rhizaria;__Cercozoa;__Silicofilosea;__Chlorarachniophyta |
| Eukaryota;__SAR;__Rhizaria;__Cercozoa;__Silicofilosea;__Euglyphida |
| Eukaryota;__SAR;__Rhizaria;__Cercozoa;__Silicofilosea;__Marimonadida |
| Eukaryota;__SAR;__Rhizaria;__Cercozoa;__Silicofilosea;__Spongomonadida |
| Eukaryota;__SAR;__Rhizaria;__Cercozoa;__Silicofilosea;__Thaumatomonadida |
| Eukaryota;__SAR;__Rhizaria;__Cercozoa;__Silicofilosea;__p15D09 |
| Eukaryota;__SAR;__Rhizaria;__Cercozoa;__Thecofilosea;__BOLA322 |
| Eukaryota;__SAR;__Rhizaria;__Cercozoa;__Thecofilosea;__Cryomonadida |
| Eukaryota;__SAR;__Rhizaria;__Cercozoa;__Thecofilosea;__Ebriacea |
| Eukaryota;__SAR;__Rhizaria;__Cercozoa;__Thecofilosea;__NOR26 |
| Eukaryota;__SAR;__Rhizaria;__Cercozoa;__Thecofilosea;__Phaeodarea |
| Eukaryota;__SAR;__Rhizaria;__Cercozoa;__Thecofilosea;__WHOI-LI1-14 |
| Eukaryota;__SAR;__Rhizaria;__Cercozoa;__Thecofilosea;__uncultured |
| Eukaryota;__SAR;__Rhizaria;__Endomyxa;__Ascetosporea;__Haplosporida |
| Eukaryota;__SAR;__Rhizaria;__Foraminifera;__Globothalamea;__Rotaliida |
| Eukaryota;__SAR;__Rhizaria;__Radiolaria;__Acantharia;__Chaunocanthida |
| Eukaryota;__SAR;__Rhizaria;__Radiolaria;__Acantharia;__Symphyacanthida |
| Eukaryota;__SAR;__Stramenopiles;__Bicosoecida;__Bicosoecidae;__Bicosoeca |
| Eukaryota;__SAR;__Stramenopiles;__Bicosoecida;__Cafeteriidae;__BCI5F15RM3E05 |
| Eukaryota;__SAR;__Stramenopiles;__Bolidomonas;__Bolidomonas_pacifica;Other |
| Eukaryota;__SAR;__Stramenopiles;__C2-E039;__uncultured_stramenopile;Other |
| Eukaryota;__SAR;__Stramenopiles;__Chrysophyceae;__CCMP1899;__Chrysophyceae_sp._I76 |
| Eukaryota;__SAR;__Stramenopiles;__Chrysophyceae;__Chromulinales;__JBNA46 |
| Eukaryota;__SAR;__Stramenopiles;__Chrysophyceae;__Chromulinales;__LG31-02 |
| Eukaryota;__SAR;__Stramenopiles;__Chrysophyceae;__Chromulinales;__uncultured_marine_eukaryote |
| Eukaryota;__SAR;__Stramenopiles;__Chrysophyceae;__E222;__uncultured_marine_eukaryote |
| Eukaryota;__SAR;__Stramenopiles;__Chrysophyceae;__E222;__uncultured_marine_picoeukaryote |
| Eukaryota;__SAR;__Stramenopiles;__Chrysophyceae;__LG01-09;__Clade_C |
| Eukaryota;__SAR;__Stramenopiles;__Chrysophyceae;__LG01-09;__uncultured_marine_chrysophyte |
| Eukaryota;__SAR;__Stramenopiles;__Chrysophyceae;__LG21-05;__uncultured_eukaryote |
| Eukaryota;__SAR;__Stramenopiles;__Chrysophyceae;__Ochromonadales;__Chrysonephele |
| Eukaryota;__SAR;__Stramenopiles;__Chrysophyceae;__Ochromonadales;__Epipyxis |
| Eukaryota;__SAR;__Stramenopiles;__Chrysophyceae;__Ochromonadales;__Ochromonas |
| Eukaryota;__SAR;__Stramenopiles;__Chrysophyceae;__Ochromonadales;__Paraphysomonas |
| Eukaryota;__SAR;__Stramenopiles;__Diatomea;__Bacillariophytina;__Bacillariophyceae |
| Eukaryota;__SAR;__Stramenopiles;__Diatomea;__Bacillariophytina;__Mediophyceae |
| Eukaryota;__SAR;__Stramenopiles;__Diatomea;__Coscinodiscophytina;__Melosirids |
| Eukaryota;__SAR;__Stramenopiles;__Diatomea;__Coscinodiscophytina;__Rhizosolenids |
| Eukaryota;__SAR;__Stramenopiles;__Eustigmatales;__Vischeria;__Vischeria_punctata |
| Eukaryota;__SAR;__Stramenopiles;__Incertae_Sedis;__Pirsonia;__uncultured_eukaryote |
| Eukaryota;__SAR;__Stramenopiles;__Incertae_Sedis;__Wobblia;__marine_gliding_biciliate_TCS-2004 |
| Eukaryota;__SAR;__Stramenopiles;__Labyrinthulomycetes;__D2P04F01;__uncultured_marine_eukaryote |
| Eukaryota;__SAR;__Stramenopiles;__Labyrinthulomycetes;__Labyrinthula;__uncultured_eukaryote |
| Eukaryota;__SAR;__Stramenopiles;__Labyrinthulomycetes;__PW19;__Oblongichytrium |
| Eukaryota;__SAR;__Stramenopiles;__Labyrinthulomycetes;__Thraustochytriaceae;__Aplanochytrium |
| Eukaryota;__SAR;__Stramenopiles;__Labyrinthulomycetes;__Thraustochytriaceae;__Diplophrys |
| Eukaryota;__SAR;__Stramenopiles;__Labyrinthulomycetes;__Thraustochytriaceae;__E170 |
| Eukaryota;__SAR;__Stramenopiles;__Labyrinthulomycetes;__Thraustochytriaceae;__Thraustochytrium |
| Eukaryota;__SAR;__Stramenopiles;__Labyrinthulomycetes;__Thraustochytriaceae;__Ulkenia |
| Eukaryota;__SAR;__Stramenopiles;__Labyrinthulomycetes;__Thraustochytriaceae;__uncultured_eukaryote |
| Eukaryota;__SAR;__Stramenopiles;__MAST-1;__MAST-1_C;__uncultured_eukaryote |
| Eukaryota;__SAR;__Stramenopiles;__MAST-12;__uncultured_eukaryote;Other |
| Eukaryota;__SAR;__Stramenopiles;__MAST-12;__uncultured_marine_picoeukaryote;Other |
| Eukaryota;__SAR;__Stramenopiles;__MAST-3;__eukaryote_marine_clone_ME1-18;Other |
| Eukaryota;__SAR;__Stramenopiles;__MAST-3;__uncultured_eukaryote;Other |
| Eukaryota;__SAR;__Stramenopiles;__MAST-3;__uncultured_marine_eukaryote;Other |
| Eukaryota;__SAR;__Stramenopiles;__MAST-3;__uncultured_stramenopile;Other |
| Eukaryota;__SAR;__Stramenopiles;__MAST-4;__uncultured_marine_eukaryote;Other |
| Eukaryota;__SAR;__Stramenopiles;__MAST-4;__uncultured_stramenopile;Other |
| Eukaryota;__SAR;__Stramenopiles;__MAST-6;__eukaryote_marine_clone_ME1-24;Other |
| Eukaryota;__SAR;__Stramenopiles;__MAST-6;__uncultured_stramenopile;Other |
| Eukaryota;__SAR;__Stramenopiles;__MAST-7;__eukaryote_marine_clone_ANT12-10;Other |
| Eukaryota;__SAR;__Stramenopiles;__MAST-9;__Pirsonia;__Pirsonia_verrucosa |
| Eukaryota;__SAR;__Stramenopiles;__MAST-9;__uncultured_stramenopile;Other |
| Eukaryota;__SAR;__Stramenopiles;__Ochromonadaceae_environmental_sample;Other;Other |
| Eukaryota;__SAR;__Stramenopiles;__Peronosporomycetes;__Haliphthoros;__Haliphthoros_sp._NJM_0034 |
| Eukaryota;__SAR;__Stramenopiles;__Peronosporomycetes;__Halophytophthora;__uncultured_eukaryote |
| Eukaryota;__SAR;__Stramenopiles;__Peronosporomycetes;__Phytophthora;__Plasmopara |
| Eukaryota;__SAR;__Stramenopiles;__Peronosporomycetes;__Pythium;__Pythiaceae_sp._PHY2 |
| Eukaryota;__SAR;__Stramenopiles;__Raphidophyceae;__E133;__uncultured_stramenopile |
| Eukaryota;__SAR;__Stramenopiles;__Synurales;__Synura;__Synura_sp._HCB-2005 |
| Eukaryota;__SAR;__Stramenopiles;__Synurales;__Tessellaria;__Tessellaria_volvocina |
| Eukaryota;__SAR;__Stramenopiles;__TKR07M.92;__uncultured_eukaryote;Other |

**Table S4.** PERMANOVA tests to determine differences between experimental warming treatments at different taxonomic scales. Variability in multivariate community structure was examined with PERMANOVA for communities of **(a)** bacteria **(b)** protists and **(c)** metazoans. The abundances of response variables were square-root transformed prior to constructing a Bray-Curtis similarity matrix. Analyses were based on 999 unique unrestricted permutations. Where significant differences were detected (at P<0.05, shown in bold) pairwise comparisons were conducted to determine which treatment levels differed from one another. The degrees of freedom associated with each test are shown in subscripted parentheses. Bacteria and metazoans were aggregated according to the Linnaean classification system, protists were aggregated according to the taxonomic hierarchy presented in Table S3.

**Response variable SS MS *F P* Pairwise tests**

**a**: Bacteria _(2,11)_

Genus 1519 759 2.10 **0.001** C≠Δ3&Δ5, Δ3=Δ5

Family 1179 589 2.05 **0.001** C≠Δ3&Δ5, Δ3=Δ5

Order 778 389 2.11 **0.003** C≠Δ3&Δ5, Δ3=Δ5

Class 534 267 1.92 **0.019** C≠Δ3, Δ5=C&Δ3

Phylum 343 172 2.19 **0.005** C≠Δ3, Δ5=C&Δ3

**b**: Protists _(2,10)_

Level 5 5797 2898 2.24 **0.001** C≠Δ3&Δ5, Δ3=Δ5

Level 4 4573 2286 2.30 **0.003** C≠Δ3&Δ5, Δ3=Δ5

Level 3 3080 1540 2.12 **0.012** C≠Δ3, Δ5=C&Δ3

Level 2 1767 883 2.16 **0.030** C≠Δ3, Δ5=C&Δ3

Level 1 811 405 1.41 0.177 n/a

**c**: Metazoans _(2,27)_

Genus 4592 2296 4.35 **0.001** C≠Δ3&Δ5, Δ3=Δ5

Family 4551 2275 4.42 **0.001** C≠Δ3&Δ5, Δ3=Δ5

Order 3089 1544 5.80 **0.001** C≠Δ3&Δ5, Δ3=Δ5

Class 3138 1569 7.31 **0.002** C≠Δ3&Δ5, Δ3=Δ5

Phylum 3138 1569 7.31 **0.002** C≠Δ3&Δ5, Δ3=Δ5

References

Amaral-Zettler, L. A., E. A. McCliment, H. W. Ducklow, and S. M. Huse. 2009. A method for studying protistan diversity using massively parallel sequencing of V9 hypervariable regions of small-subunit ribosomal RNA genes. PLoS ONE 4:e6372.

Caporaso, G., J. Kuczynski, J. Stombaugh, K. Bittinger, F. Bushman, E. Costello, N. Fierer, A. Peña, J. Goodrich, J. Gordon, G. Huttley, S. Kelley, D. Knights, J. Koenig, R. Ley, C. Lozupone, D. McDonald, B. Muegge, M. Pirrung, J. Reeder, J. Sevinsky, P. Turnbaugh, W. Walters, J. Widmann, T. Yatsunenko, J. Zaneveld, and R. Knight. 2010. QIIME allows analysis of high-throughput community sequencing data. Nature Methods 7:335-336.

Caporaso, J. G., C. L. Lauber, W. A. Walters, D. Berg-Lyons, C. A. Lozupone, P. J. Turnbaugh, N. Fierer, and R. Knight. 2011. Global patterns of 16S rRNA diversity at a depth of millions of sequences per sample. Proceedings of the National Academy of Sciences 108:4516-4522.

DeSantis, T. Z., P. Hugenholtz, N. Larsen, M. Rojas, E. L. Brodie, K. Keller, T. Huber, D. Dalevi, P. Hu, and G. L. Andersen. 2006. Greengenes, a chimera-checked 16S rRNA gene database and workbench compatible with ARB. Applied Environmental Microbiology 72:5069-5072.

Suzuki, M. T., L. T. Taylor, and E. F. DeLong. 2000. Quantitative analysis of small-subunit rRNA genes in mixed microbial populations via 5′-nuclease assays. Applied and Environmental Microbiology 66:4605-4614.

Taylor, J. D., S. D. Cottingham, J. Billinge, and M. Cunliffe. 2014. Seasonal microbial community dynamics correlate with phytoplankton-derived polysaccharides in surface coastal waters. ISME Journal 8:245-248.

Taylor, J. D., and M. Cunliffe. 2015. Polychaete burrows harbour distinct microbial communities in oil-contaminated coastal sediments. Environmental Microbiology Reports 7:606-613.

Zhu, F., R. Massana, F. Not, D. Marie, and D. Vaulot. 2005. Mapping of picoeukaryotes in marine ecosystems with quantitative PCR of the 18S rRNA gene. FEMS Microbiology Ecology 52:79-92.
